# Ohio State Homœopathic Society

**Published:** 1884-05

**Authors:** H. E. Beebe

**Affiliations:** Secretary


### HOMŒOPATHIC MEDICAL SOCIETY OF OHIO.

DEAR DOCTOR: The Twentieth Annual Session of the Homœopathic Medical Society of Ohio will be held in Cleveland, May 13th and 14th, 1884. We would like to see every homœopathic physician in the State at this meeting. There will be transacted much business of importance to us all. Are you on a bureau? If so, send your topic at once. If not on a committee, volunteer papers are very acceptable.

Will you be with us? The programme will be out in a few days.

H. E. BEEBE, *Secretary.*

